# Mono-Component Feature Extraction for Condition Assessment in Civil Structures Using Empirical Wavelet Transform

**DOI:** 10.3390/s19194280

**Published:** 2019-10-02

**Authors:** Yun-Xia Xia, Yun-Lai Zhou

**Affiliations:** 1 School of Civil Engineering, Qingdao University of Technology, Qingdao 266033, China; xiayunxia@qut.edu.cn; 2 Faculty of Engineering, Universidade Lusófona, 1749-024 Lisbon, Portugal

**Keywords:** signal processing, empirical wavelet transform, structural health monitoring, feature extraction, civil structures

## Abstract

This paper proposes a methodology to process and interpret the complex signals acquired from the health monitoring of civil structures via scale-space empirical wavelet transform (EWT). The FREEVIB method, a widely used instantaneous modal parameters identification method, determines the structural characteristics from the individual components separated by EWT first. The scale-space EWT turns the detecting of the frequency boundaries into the scale-space representation of the Fourier spectrum. As well, to find meaningful modes becomes a clustering problem on the length of minima scale-space curves. The Otsu’s algorithm is employed to determine the threshold for the clustering analysis. To retain the time-varying features, the EWT-extracted mono-components are analyzed by the FREEVIB method to obtain the instantaneous modal parameters and the linearity characteristics of the structures. Both simulated and real SHM signals from civil structures are used to validate the effectiveness of the present method. The results demonstrate that the proposed methodology is capable of separating the signal components, even those closely spaced ones in frequency domain, with high accuracy, and extracting the structural features reliably.

## 1. Introduction

The data in Structural Health Monitoring (SHM) from civil structures contains essential information on their condition. Instantaneous features shall be desirable to be extracted for further structural condition assessment. However, under operating condition, the dynamic responses of civil structures are usually stored in time domain and are non-stationary because of complex excitation. They include complex components induced by distinct loads and intricate load-structure interactions. In addition, the acquired data includes unavoidable noises, spikes and trends, further challenging the extraction of useful information.

Time-frequency (TF) approaches were developed to obtain the instantaneous features from time-varying signals. These methods can provide information of signals in both time and frequency domains, becoming more competitive to process SHM data. Various TF methods including Short Time Fourier Transform (STFT) [1], Wigner-Ville Distribution (WVD) [2], Wavelet Transform (WT) [3], and Empirical Mode Decomposition (EMD) [4] were raised. Even these methods can determine some useful results, their limitations are also clear. For instance, the window length fixes the spectral resolution of the STFT. Despite the WVD can provide a good resolution, the cross-term inference restricts its application. With the merit of multi-resolution, the WT has been one of the most widely used signal processing methods in recent years [5,6,7]. In civil engineering, WT has served as an important tool for signal processing, system identification and damage detection of structures [8,9,10,11]. However, the wavelet basis selection restricted its effect. WT sometimes cannot provide frequency resolution precisely enough to describe the time variations of signal frequencies. To improve the time and frequency resolution of WT, Daubechies et al. proposed the synchrosqueezed WT (SWT), a combination of wavelet analysis and reallocation method [12]. SWT focuses on utilizing the output of the classic WT, the wavelet basis is yet predefined and invariant. EMD does not use any prescribed function basis, and it is self-adaptive to the analyzed signal. Therefore, EMD is highly adaptable and can extract the non-stationary components from the given signals. EMD has been extensively used for damage detection in SHM [13,14,15]. To solve the mode mixing problem in EMD, the ensemble EMD (EEMD) was further developed by Wu and Huang [16]. However, EMD relied on an ad-hoc process difficult to be mathematically modeled, sometimes it might be challenging to understand the physical implications of the EMD results.

In contrast, the empirical wavelet transform (EWT) [17] proposed by Gilles (2013) combines the advantages of WT and EWD. This method not only can decompose signals adaptably with high TF resolution, but also have a consolidated mathematical foundation on WT. It is highly favorable for the processing and interpretation of non-stationary and complex signals. This method has been applied to process signals in various areas like biomedical, wind, earthquake, and mechanical engineering [18,19,20,21,22,23,24,25,26,27]. Kedadouche et al. [28] conducted a comparative study between EMD and EWT, showing that EWT outperforms EMD on mode estimation and computation time.

For processing real SHM signals of civil structures with the original EWT [17], spectrum segmentation shall be difficult due to noises, leading to irrelevant modes to the structural condition and false modes are difficult to interpret. The Gilles’ approach detects the boundaries to build the wavelet filter bank in the Fourier spectrum via the identification of local maxima and minima. The Fourier spectrum is very susceptible to noises, leading to spurious local maxima. Another limitation of the original EWT method is a predefined boundary number.

One way to improve the original EWT is employing spectrum other than the Fourier one, such as the standardized autoregression power spectrum [29], and the pseudo-spectrum obtained by the multiple signal classification approach [30,31]. However, few studies discuss if meaningful modes could be eliminated by these spectra. Superior EWT can also resort to boundary detection approaches more competent than the one used by Gilles [17].

This paper adopts a scale-space EWT based on scale-space approach [32], a combination of EWT and Otsu’s method, which can automatically detect the useful modes in the scale-space representation the Fourier spectrum. Finding modes is converted to a clustering problem on the length of minima scale-space curves. The key point is to determine a threshold automatically. The probabilistic approach, Otsu’s method, and *k*-Means algorithm are three ways to find the threshold.

The estimation of the free decaying vibration function subjected to ambient vibrations will correspond to each mode by approaches such as Random Decrement Technique (RDT) [29] and the Natural Excitation Technique (NExT) [31]. The natural frequencies and damping ratios of the structures are further identified. However, the parameters obtained in this manner are constant in time, implying that the instantaneous features have been eliminated.

The FREEVIB method, a nonparametric approach proposed by Feldman [33] for the single-degree-of-freedom (SDOF) system under free vibration, can identify the instantaneous linear and nonlinear modal parameters. For a multi-degree-of-freedom (MDOF) system, the vibration signals can be decomposed into mono-components first. If the subsystems are uncoupled, the structural parameters can be subsequently identified using the method for an SDOF structure.

This paper proposes an EWT-based methodology to extract mono-component features from SHM signals in the condition assessment for civil structures. Signals are decomposed using the scale-space EWT first. For structural feature interpretation, the FREEVIB method is then applied to the separated mono-components to identify the instantaneous modal parameters and the linearity characteristics. Simulated signals with different levels of noise and real SHM signals from two civil structures, namely, a high-rise building and a footbridge, are used to demonstrate the effectiveness of the proposed signal processing procedure. The usefulness and accuracy of the scale-space EWT in signal decomposition is validated by the synthetic signals. To show the advantages of the scale-space EWT, the TF representations are compared with those from SWT and EMD. In the experimental study, the extracted instantaneous structural features are affirmed by comparing with results of the previous studies.

## 2. Empirical Wavelet Transform

The EWT aims to extract the individual modes from a signal by an adaptively designed wavelet filter bank. The modes extracted by EWT are amplitude modulated-frequency modulated (AM-FM) signals that have a compact support Fourier spectrum [17]. Separating different modes equals dividing the Fourier spectrum first and then to perform WT using the empirical wavelets constructed based on the detected supports.

Fast Fourier Transform (FFT) is implemented to the signal *s*(*t*) to obtain the frequency spectrum *s*(*ω*). Segmenting the Fourier spectrum is the crucial step for the adaptability of EWT. The local maxima of *s*(*ω*) is estimated first. Then, the Fourier axis is segmented to individual portions corresponding to different modes that are centered around a local maximum. The number of continuous segments the Fourier axis divided into is denoted as *N*. The limits between each segment are denoted as *ω_i_* (*ω*_0_ = 0, and *ω_N_* = *π*), and each segment is denoted as *Λ_i_* = [*ω*_*i*−1_, *ω_i_*]. A transition phase *T_i_* of width 2*τ_i_* is defined surrounding each *ω_i_*. The simplest choice of *τ_i_* is

(1) $$\tau_{i}=\gamma \omega_{i},\;\;\;0<\gamma <1$$

Similar to constructing Littlewood-Paley and Meyer’s wavelets [3], the empirical scaling function and the empirical wavelets can be defined by the following expressions of Equations (2) and (3), respectively.

(2) $${\hat{\phi }}_{i}(\omega )=\left\{ \begin{array}{cc} 1 & \left|\omega \right|\leq (1-\gamma )\omega_{i}\\ \cos[\frac{\pi }{2}\beta (\frac{1}{2\gamma \omega_{i}}(\left|\omega \right|-(1-\gamma )\omega_{i}))] & (1-\gamma )\omega_{i}\leq \left|\omega \right|\leq (1+\gamma )\omega_{i}\\ 0 & \mathrm{otherwise} \end{array}\right.$$

and

(3) $${\hat{\psi }}_{i}(\omega )=\left\{ \begin{array}{cc} 1 & (1+\gamma )\omega_{i}\leq \left|\omega \right|\leq (1-\gamma )\omega_{i+1}\\ \cos[\frac{\pi }{2}\beta (\frac{1}{2\gamma \omega_{i+1}}(\left|\omega \right|-(1-\gamma )\omega_{i+1}))] & (1-\gamma )\omega_{i+1}\leq \left|\omega \right|\leq (1+\gamma )\omega_{i+1}\\ \sin[\frac{\pi }{2}\beta (\frac{1}{2\gamma \omega_{i+1}}(\left|\omega \right|-(1-\gamma )\omega_{i}))] & (1-\gamma )\omega_{i}\leq \left|\omega \right|\leq (1+\gamma )\omega_{i}\\ 0 & \mathrm{otherwise} \end{array}\right.$$

*β*(*x*) is an arbitrary polynomial function *C^k^* ([0, 1])

(4) $$\beta (x)=\left\{ \begin{array}{l} 0\;\;\;\;x\leq 0\\ 1\;\;\;\;\;x\geq 0 \end{array}\right.\;\;\;\;\;\;\;\;\;\beta (x)+\beta (1-x)=1\;\;\;\forall x\in [0,1]$$

After building a tight frame set of empirical wavelets, the EWT can be defined. The detailed coefficients are [17]

(5) $$W_{s}^{\varepsilon }(i,t)=<s,\psi_{i}>=\int s(\tau )\bar{\psi_{i}(\tau -t)}\;d\tau ={\left(\hat{s}(\omega )\bar{{\hat{\psi }}_{i}(\omega )}\right)}^{\vee }$$

and the approximation coefficients are

(6) $$W_{s}^{\varepsilon }(0,t)=<s,\phi_{1}>=\int s(\tau )\bar{\phi_{1}(\tau -t)}\;d\tau ={\left(\hat{s}(\omega )\bar{{\hat{\phi }}_{1}(\omega )}\right)}^{\vee }$$

The signal is reconstructed by

(7) $$s(t)=W_{s}^{\varepsilon }(0,t)\times \phi_{1}(t)+\sum_{i=1}^{N}W_{s}^{\varepsilon }(i,t)\times \psi_{i}(t)={\left({\hat{W}}_{s}^{\varepsilon }(0,\omega )\times {\hat{\phi }}_{1}(\omega )+\sum_{i=1}^{N}{\hat{W}}_{s}^{\varepsilon }(i,\omega )\times {\hat{\psi }}_{i}(\omega )\right)}^{\vee }$$

The empirical mode *s_k_* is given by

(8) $$s_{0}(t)=W_{s}^{\varepsilon }(0,t)\times \phi_{1}(t)$$

(9) $$s_{k}(t)=W_{s}^{\varepsilon }(k,t)\times \psi_{k}(t)$$

## 3. Methodology

**Figure 1:**
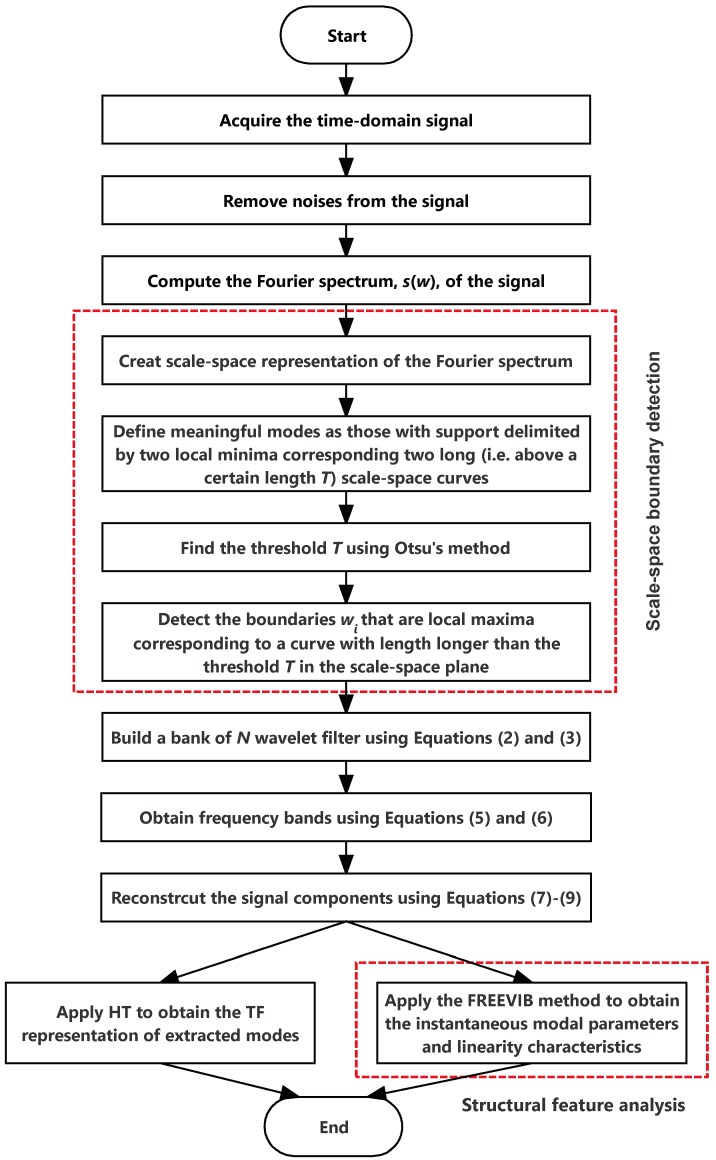
Flowchart of the proposed signal processing methodology.

The flowchart of the methodology is shown in Figure 1. The steps are illustrated as follows.

### 3.1. Scale-Space Boundary Detection

As the most important step in EWT, boundary detection to build the wavelet filter bank provides adaptability to the analyzed signal. Gilles [17] estimated the boundaries based on the local maxima and minima of the signal’s Fourier spectrum. However, the Fourier spectrum is very sensitive to noises, as is usual the case for SHM signals of civil structures. These noises may produce redundant local maxima, leading to false boundaries. The Gilles’ method requires the predefined frequency-band number. Other spectra immune to noises [29,30] can be employed, but special attention should be paid to the probability of missing useful modes. This study takes an alternative way, to use a different method other than the original local-maxima-minima one to segment the Fourier spectrum. The scale-space approach, which automatically detects meaningful modes in a spectrum based on the behavior of local minima in a scale-space representation [32], is employed. Finding meaningful modes is equivalent to a binary clustering problem on the length of minima scale-space curves. To reduce the influence of noises, a wavelet-based approach [7] is employed for denoising before the EWT procedure.

#### 3.1.1. Scale-Space Representation of a Spectrum

Let function *f*(*ω*) define over an interval [0, *ω_max_*]. Its discrete scale-space representation is defined as

(10) $$L(m,\sigma )=\sum_{n=-M}^{n=+M}f(m-n)g(n;\sigma )$$

where

(11) $$g(n;\sigma )=\frac{1}{\sqrt{2\pi \sigma }}e^{-n^{2}/2\sigma }$$

which is a sampled Gaussian kernel, *M* is large enough so that the approximation error of the Gaussian is negligible. The scale parameter *σ* is sampled in the following manner

(12) $$\sqrt{\sigma }=k\sqrt{\sigma_{0}}$$

where *k* = 1, …, *k*_max_ are integers, $\sqrt{\sigma_{0}}$ is set to be 0.5, and $\sqrt{\sigma_{\max}}$ equals *ω_max_*.

#### 3.1.2. Definition of Meaningful Modes

The number of minima with respect to *ω* of *L*(*ω*, *σ*) is a decreasing function of the scale parameter *σ* [32]. In the scale-space plane, a curve is produced by each initial minima. Let us denote the number of initial minima as *N*_0_, and the ‘scale-space curve’ defined as *C_i_* (*i* ∈ [1, *N*_0_]), with a length of *L_i_*. *L_i_* indicates the life span of the minimum *i*. A mode in a spectrum is defined as meaningful if its support is delimited by two local minima corresponding to two scale-space curves *C_i_* above a certain length *T* [32]. Consequently, detecting meaningful modes is a two-class clustering problem on {*L_i_*}*_i_*_∈__[1, *N*_0_]_. The key point is to automatically determine a threshold *T*.

#### 3.1.3. Determination of Threshold

The Otsu’s method separates a spectrum into two classes of *C*_1_ and *C*_2_, and finds *T* that maximizes the between class variance

(13) $$\sigma_{B}^{2}=W_{1}W_{2}{\left(\mu_{1}-\mu_{2}\right)}^{2}$$

where

(14) $$W_{r}=\frac{1}{n}\sum_{j\in H_{r}}C(j)$$

and

(15) $$\mu_{r}=\frac{1}{n}\sum_{j\in H_{r}}jC(j)$$

Details of the Otsu’s method can be found in [34].

### 3.2. Time-Frequency Representation of Extracted Modes

The modes extracted by the EWT are AM-FM signals *s_j_*(*t*) = *S_j_*(*t*)cos(*φ_j_*(*t*)) (*j* = 0, 1, …, *N*). Following Hilbert-Huang transform (HHT), the HT of a function *s_j_*(*t*) is defined as

(16) $$H_{sj}(t)=\frac{1}{\pi }p.v.\int_{-\infty }^{+\infty }\frac{s_{j}(\tau )}{t-\tau }d\tau$$

where *p*.*v*. represents the Cauchy principle value.

The analytical form *s_ja_*(*t*) of *s_j_*(*t*) can be derived by the HT

(17) $$s_{ja}(t)=s_{j}(t)+iH_{sj}(t)$$

In AM-FM signals the HT provides

(18) $$s_{ja}(t)=S_{j}(t)e^{i\varphi_{j}(t)}$$

where the instantaneous amplitude *S_j_*(*t*) and frequency *φ_j_′*(*t*) can be extracted. The TF representation of the signal is obtained by plotting each curve *φ_j_′*(*t*) with intensity of *S_j_*(*t*) in the TF plane. The time varying of the frequency and amplitude of each mode can be observed from this TF representation.

### 3.3. Structural Feature Analysis using Mono-Component

#### 3.3.1. Modal Characteristics

For a time-varying SDOF structure under free vibration, if its parameters vary slower than the dynamic response, both the natural frequency *ω*_0_(*t*) and the damping coefficient *h*_0_(*t*) are slowly varying functions of time. They can be evaluated by [33]

(19) $$\omega_{0}^{2}(t)=\omega^{2}-\frac{\ddot{A}}{A}+2\frac{{\dot{A}}^{2}}{A^{2}}+\frac{\dot{A}\dot{\omega }}{A\omega }$$

and

(20) $$h_{0}(t)=-\frac{\dot{A}}{A}-\frac{\dot{\omega }}{2\omega }$$

where *A* and *ω* are the instantaneous amplitude and frequency of the response, respectively. When it comes to a time-varying SDOF system under forced vibration, the instantaneous frequency of the vibration signal is

(21) $$\omega^{2}(t)=\omega_{0}^{2}(t)-\frac{fx+H[f]H[x]}{m[x^{2}+{\left(H[x]\right)}^{2}]}$$

For ambient vibration, the second term of Equation (21) is a zero mean fast time-varying function [35]. The instantaneous frequency and amplitude of the dynamic signals will obtain time-varying parameters.

The FREEVIB method [33] relies on HT. It can obtain the stiffness and damping characteristics, and identify the instantaneous modal parameters of free vibration SDOF systems. This method includes the following steps [36]: (1) Taking the HT of the measured dynamic responses and calculating the envelope and the instantaneous frequency; (2) identifying the instantaneous parameters; (3) low-pass filtering of the modal parameters, and scaling the smooth modal parameters; and (4) plotting the backbones of the frequency, damping curves, frequency response functions (FRF), and force static characteristics.

#### 3.3.2. Backbone and Damping Curve

From Equations (19) and (20), the instantaneous modal parameters are functions of the first and second deviations of the signal envelop and the instantaneous frequency of the dynamic response. Linking the modal frequency and the envelope gets a skeleton curve or backbone. Similarly, linking the modal damping and the envelope obtains a damping curve. Backbones and damping curves are used as a traditional instrument in nonlinear vibration analysis [36].

For small and slow nonlinear variations,

(22) $${\dot{A}}^{2}=\ddot{A}=\dot{\omega }=\dot{A}\dot{\omega }=0$$

The instantaneous modal frequency of the system will be close to the instantaneous frequency of the dynamic response, and the instantaneous damping coefficient equals the ratio between the envelope and its derivative.

## 4. Numerical Study

A synthetic signal consisting of three frequency components of 1, 3 and 6 Hz is used in this section to investigate the advantages of EWT. White Gaussian noise included into the signal to study the noise effect. The exponential function is embedded to simulate the signal attenuation with time. This simulated signal is expressed as

(23) $$\left\{ \begin{array}{l} x(t)=x_{1}(t)+x_{\mathrm{noise}}(t)\\ x_{1}(t)=6e^{-0.05t}\cos2\pi t+4e^{-0.05t}\cos6\pi t+\\ x_{noise}(t)=n(t) \end{array}\right.2e^{-0.05t}\cos12\pi t$$

where *n*(*t*) is the white Gaussian noise. High-level noises with three different signal-to-noise ratios (SNRs), i.e., −2, −6 and −10 dB, are used to test the efficacy of the scale-space EWT method.

**Figure 2:**
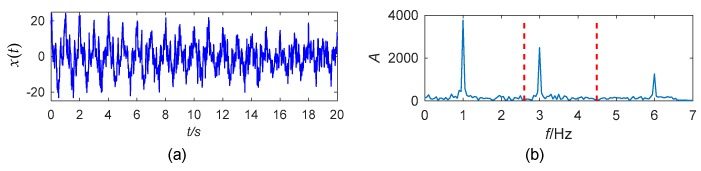
(**a**) The simulated signal; (**b**) segmentation of the Fourier spectrum.

**Figure 3:**
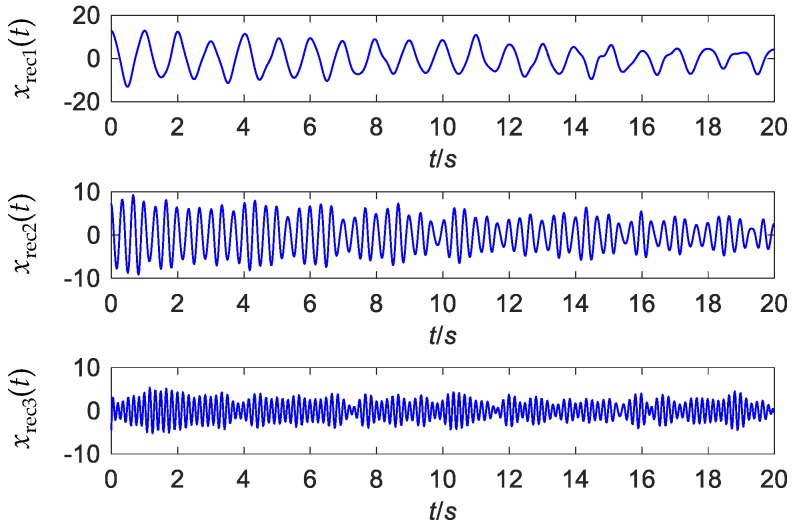
The mono-components of the simulated signal extracted by EWT.

The 20-s simulated signal with an SNR of −2 dB is shown in Figure 2a. The sampling frequency is 50 Hz. The boundaries for the spectrum segmentation are detected using the scale-space method stated in Section 3.1.1, and the result is shown in Figure 2b. The threshold for the boundary detection is calculated to be 14 by the Otsu’s method. Thus, minima scale-space curves with length longer than 14 are the boundaries. The empirical wavelets are defined according to Equations (2) and (3) based on these boundaries. Using these basis functions, WT is applied to decompose the signal. The extracted signal components, *x*_rec1_(*t*) to *x*_rec3_(*t*) are displayed in Figure 3. All the three components are extracted without redundant modes.

**Figure 4:**
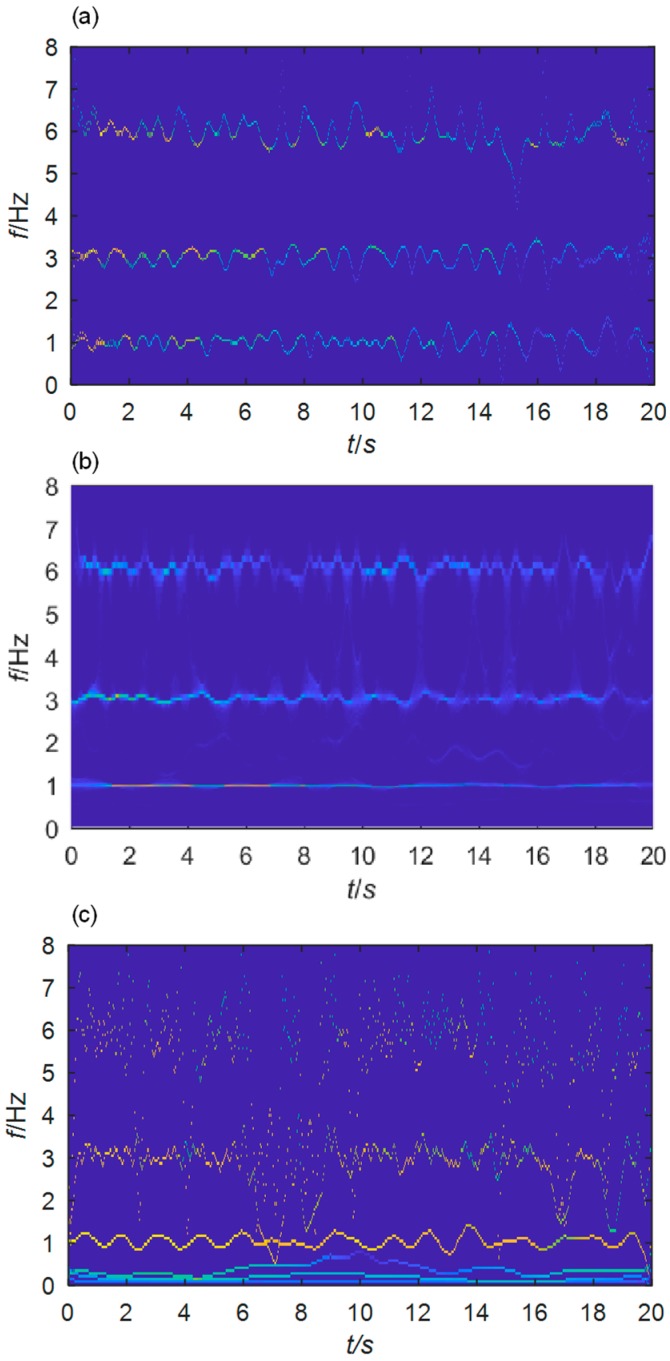
TF planes of mono-components extracted from the simulated signal by: (**a**) EWT; (**b**) SWT; (**c**) HHT.

Performing HT on each extracted component, their instantaneous frequencies are obtained. The TF plane is shown in Figure 4a, where the brightness of the instantaneous-frequency lines represents the amplitude of the corresponding components. The time varying of frequencies and amplitudes for each component can be noted. The added noise makes the frequencies fluctuate continuously. The brightness of these lines decays with time, meaning that the magnitudes of the components decrease with time. This phenomenon coincides with the fact that signal components attenuate with time due to the exponential function in Equation (23).

As comparison, SWT and EMD are also employed. In SWT, the analytical Morlet wavelet is used as the prescribed basis function. The EMD self-adaptively decomposes the signal into *N*+1 IMFs *f_k_*(*t*), which are AM-FM components

(24) $$s_{j}(t)=S_{j}(t)\cos(\varphi_{j}(t))\;\;\;\;\mathrm{where}\;S_{j}(t),\;\varphi_{j}^{\prime }(t)>0$$

under the assumption that *S_j_*(*t*) and *φ*′(*t*) vary much slower than *φ*(*t*). TF planes obtained by these two methods are shown in Figure 4b,c. The three signal components are separated by SWT with satisfactory frequency resolution. However, in the EMD results the instantaneous frequencies of the second and third components are not so legible. Redundant modes below 1 Hz are produced.

Applying the FREEVIB method to extracted signal components, the instantaneous frequencies and damping ratios for each mode are obtained. To compare the three methods of EWT, SWT, and EMD, the most probable values of these two parameters are selected as the indicators. The results of are shown in Table 1. Recognizing that the accuracy of these three methods is different, the coefficients of variation (CVs) are also listed in the table for comparison.

It can be observed that by this means all the three methods can identify the modal frequency with a value very close to the theoretical one. Larger discrepancy is found between the analyzed and theoretical damping ratios. Nonetheless, EWT performs better than the other two methods in the value estimation, especially for the third mode. As to the CVs, the EWT is superior in those of the frequency, but not so excellent in the damping ratio. It should be admitted that estimating the damping ratio accurately is difficult for all the three methods. An appropriate processing of the noises may improve the performance of the methods.

**Figure 5:**
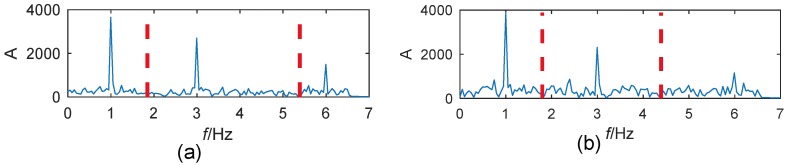
Segmentation of the Fourier spectrum for signals with an SNR of: (**a**) −6 dB; (**b**) −10 dB.

**Figure 6:**
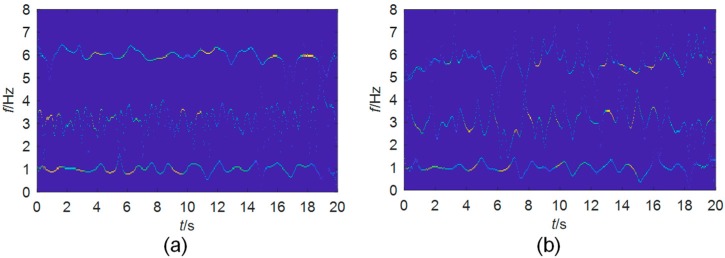
TF planes of mono-components extracted from the simulated signals with an SNR of: (**a**) −6 dB; (**b**) −10 dB.

To test the immunity of the method to noises, higher-level noises with an SNR of −6 dB and −10 dB are added to the signal, respectively. The thresholds for spectrum detection are 74 and 155 for each. The detected frequency boundaries are shown in Figure 5. As well, the TF planes for the extracted mono-components are shown in Figure 6. The accuracy of the EWT method degrades by the noises. The boundary detection shown in Figure 5 is still reliable. The instantaneous frequencies of the three modes can be well separated even when the SNR is −10 dB.

## 5. Case Study

### 5.1. A High-Rise Building

**Figure 7:**
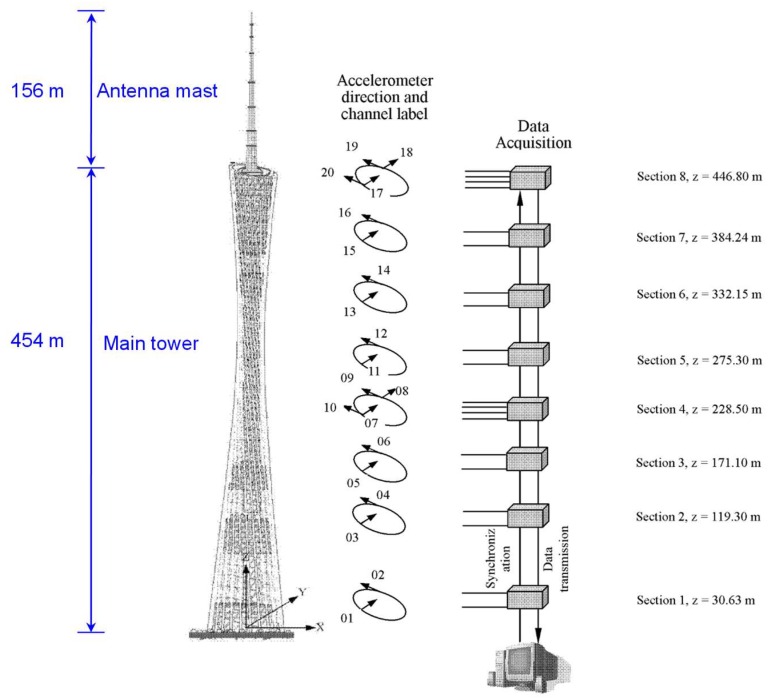
Canton tower and the layout of accelerometers (Source: http://www.zn903.com/ceyxia/benchmark/index.htm).

The Canton Tower, a 610 m high TV tower in Guangzhou, China, is another test bed for the feasibility of EWT. It is composed of a 454 m high main tower and a 156 m high antenna mast, as shown in Figure 7. The main tower has a tube-in-tube geometry consisting of a reinforced concrete inner structure and a steel lattice outer structure. Its construction was completed in May 2009.

A long-term SHM system was deployed on the tower for real-time monitoring of the structure [37]. As part of this system, 20 uniaxial accelerometers (Tokyo Sokushin AS-2000C) are installed on eight different cross-sections of the main tower (Figure 7) to measure the structural dynamic responses. Accelerometers 01, 03, 05, 07, 08, 11, 13, 15, 17, and 18, are used to collect the response in the short-axis direction, while others measure in the long-axis direction. The sampling frequency for the acceleration data is 50 Hz 24-h (18:00 p.m. 19 January 2010 to 18:00 p.m. 20 January 2010) data recorded during one construction stage are provided for a benchmark study [38].

**Figure 8:**
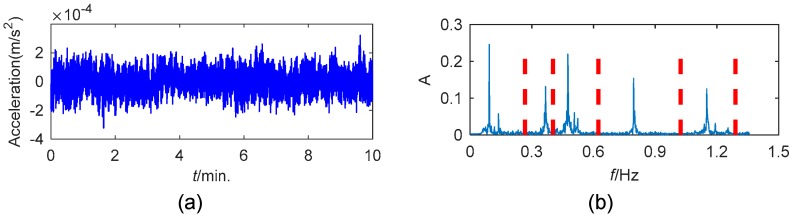
(**a**) The time history of the analyzed acceleration data from Canton Tower; (**b**) the detected boundaries in the Fourier spectrum.

**Figure 9:**
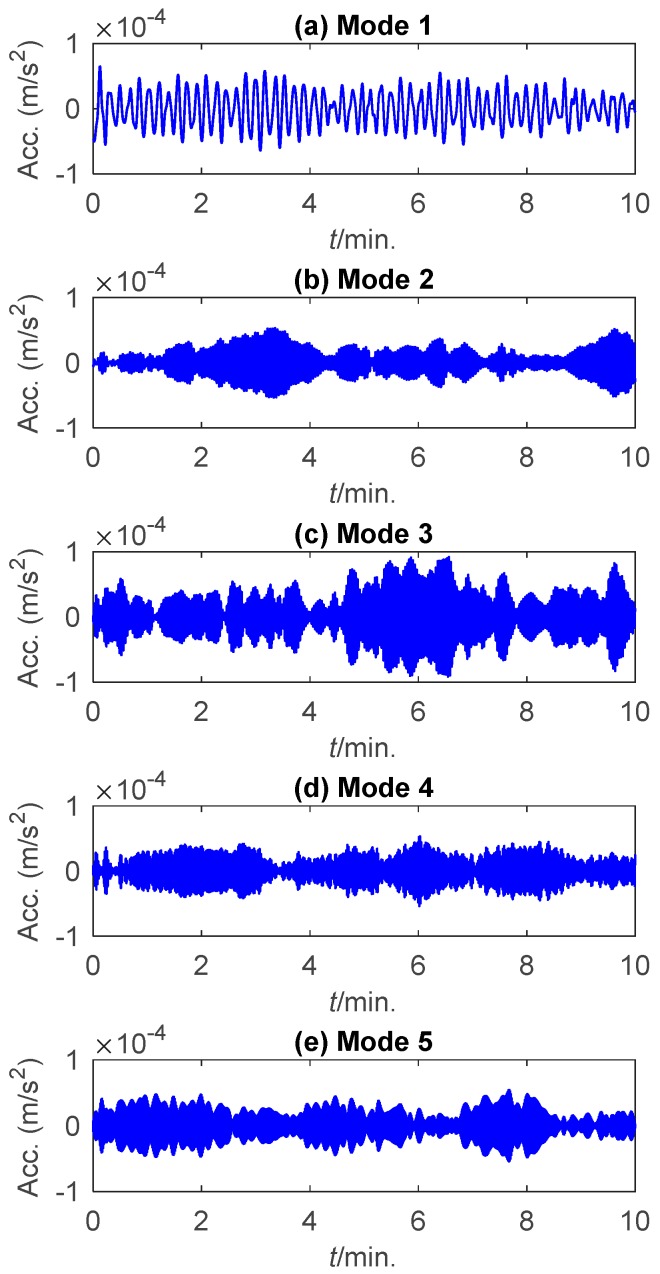
The first five components extracted from the acceleration signal of Canton Tower by EWT.

This study used the data collected by the accelerometer 11 from 05:00 a.m. to 05:10 a.m. on 20 January 2010. The time history of the data and the corresponding frequency boundaries detected by the scale-space approach are shown in Figure 8. The first five components extracted by EWT are displayed in Figure 9.

**Figure 10:**
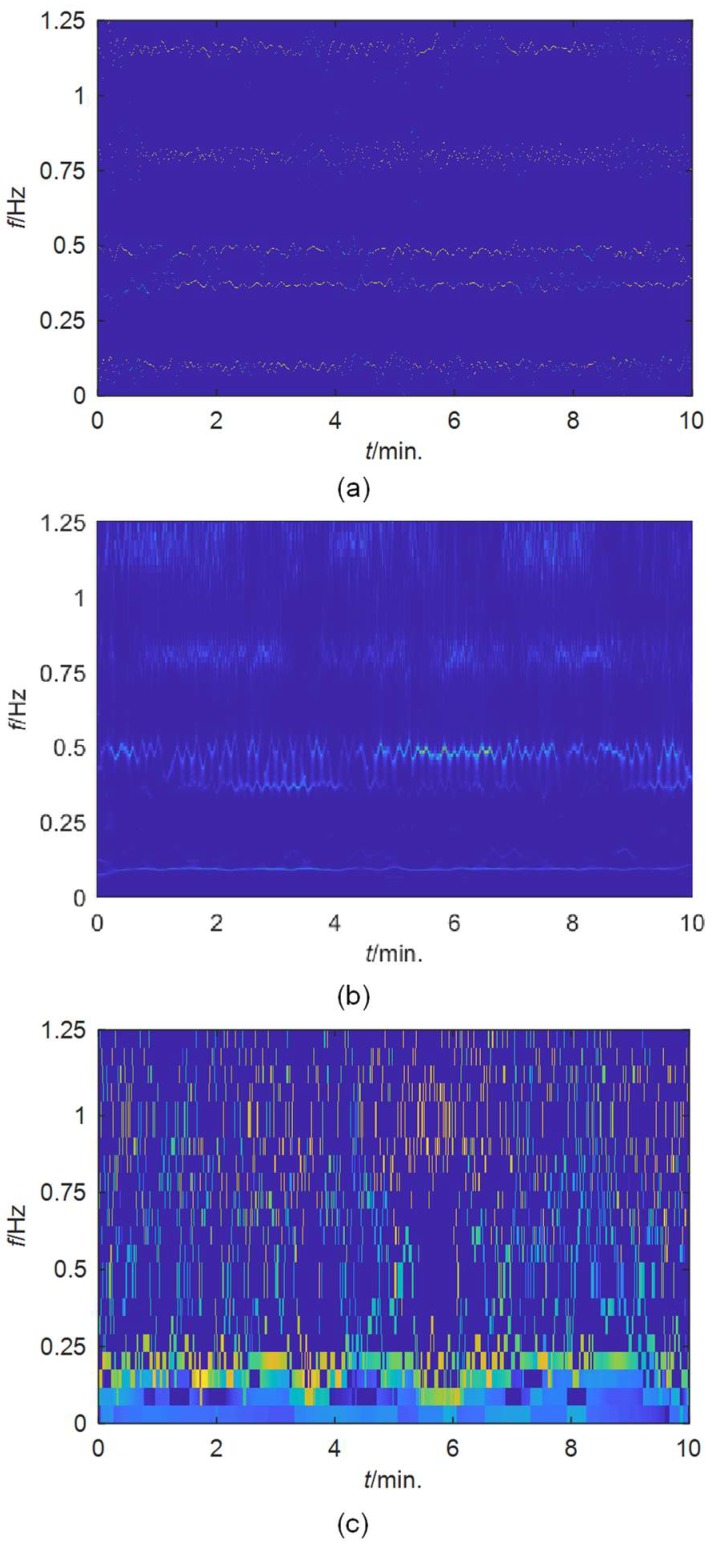
TF planes for the acceleration signal in the short-axis direction of Canton tower obtained by: (**a**) EWT; (**b**) SWT; (**c**) EMD.

Applying the FREEVIB method to the extracted mono-components, the instantaneous modal parameters of the tower are derived. The instantaneous frequencies of these modes are shown in Figure 10a. For comparison, SWT and EMD are also used to analyze this signal, the results of which are displayed in Figure 10b,c, respectively. The frequency resolution of EWT is much higher than that of the SWT and EMD. The instantaneous frequencies identified by EWT are obviously more concentrated. The second and the third modes close to each other are clearly discriminated by EWT. In contrast, the instantaneous frequencies obtained by the other two methods are relatively scattered, especially for those determined by EMD. The performance of SWT is much better than EMD, but it cannot separate the two closely spaced modes clearly as EWT.

**Figure 11:**
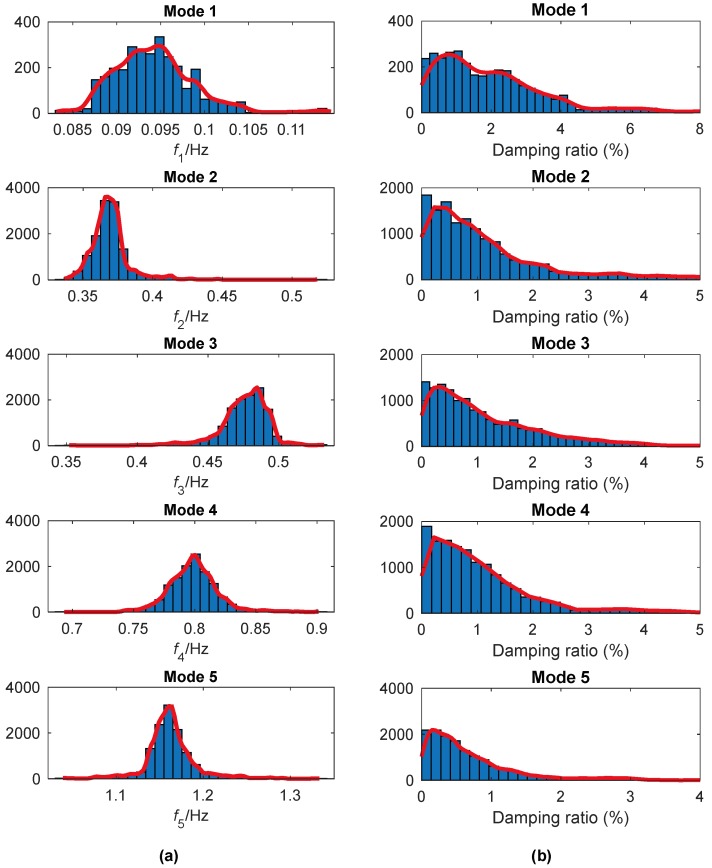
Histograms for the instantaneous modal parameters of Canton Tower identified by EWT: (**a**) Instantaneous frequency; (**b**) damping ratio.

The histograms for the instantaneous frequencies and damping ratios of the extracted modes are shown in Figure 11a,b, respectively. The most probable value for each mode is considered as the modal parameter of the tower. They are listed in Table 1 with the corresponding CVs. In [39], the CVs for the modal frequencies are less than 0.005, and those for the damping ratios are less than 0.90. The CVs in Table 2 are much larger. The main reason is that in this study they are derived directly from a randomly selected signal with a duration of only 10 min, where there may be disturbances from environment, loads and so on. In contrast, the previous study uses data segment length long enough (one-hour) to reduce the noise effects. Moreover, in this study the 24 measurements of one-hour duration were not used directly but first decomposed into 70 overlapping data sets of one-hour duration with a 20-min shift. In Table 1, another observation is that the CVs of the damping ratio are much larger than those of the modal frequency. It is mainly because that the identified damping ratio of a structure is usually not as stable as the natural frequency.

**Figure 12:**
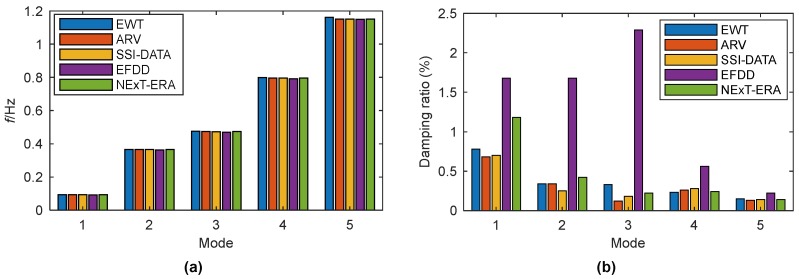
Comparison of modal parameters for Canton Tower identified by EWT, ARV, SSI-DATA, EFDD, and NExT-ERA: (**a**) Instantaneous frequency; (**b**) damping ratio.

As verification, the identified modal frequencies and damping ratios are compared with those obtained by the vector autoregressive (ARV) technique [40], the data-driven stochastic identification (SSI-DATA) method [40], the enhanced frequency domain decomposition (EFDD) algorithm [41], and an improved automatic modal identification method based on NExT-ERA [41], respectively. This comparison is shown in Figure 12 using bar plots. The modal frequencies identified by EWT agree quite well with those obtained by the other four methods. Though relatively big differences exist in the damping ratios identified by distinct methods, the values obtained from EWT have a satisfactory accordance with those determined from ARV and SSI-DATA.

**Figure 13:**
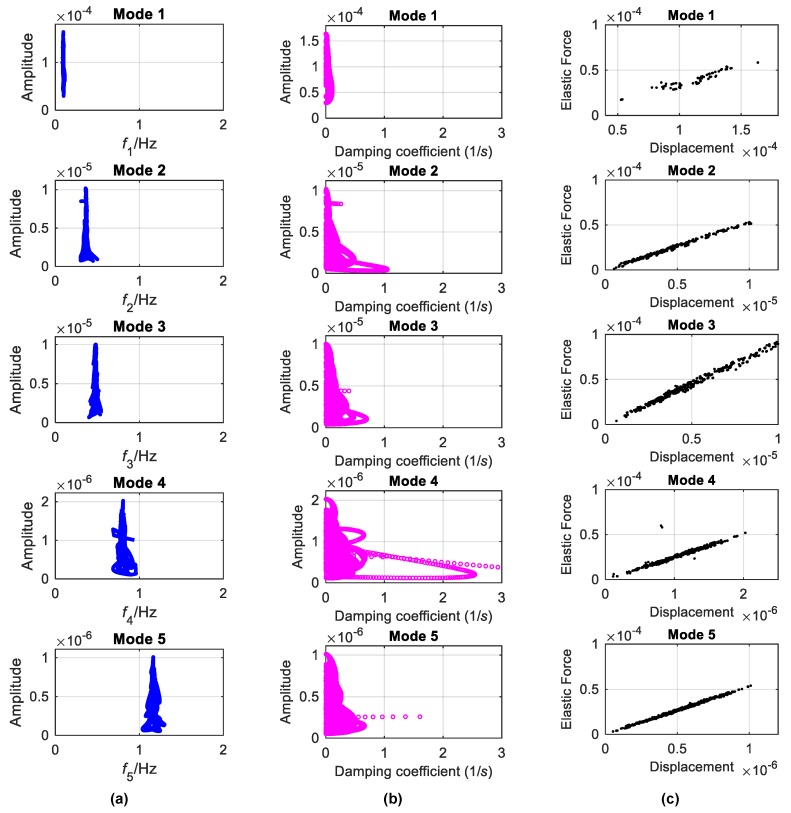
Linear characteristics identified from the signal mono-components of Canton Tower: (**a**) backbones; (**b**) damping coefficients; (**c**) elastic forces.

The backbones estimated by the FREEVIB method for each mode and the corresponding damping curves are displayed in Figure 13a,b, respectively. The identified instantaneous frequencies almost do not vary with the vibration amplitude. On the other hand, for the damping coefficients no obvious varying trends exist with the amplitude, though they are more scattered than the instantaneous frequencies. This implies that under the analyzed condition the Canton tower can be considered as a linear system. This is affirmed by the identified elastic-force characteristics illustrated in Figure 13c.

### 5.2. A Footbridge

**Figure 14:**
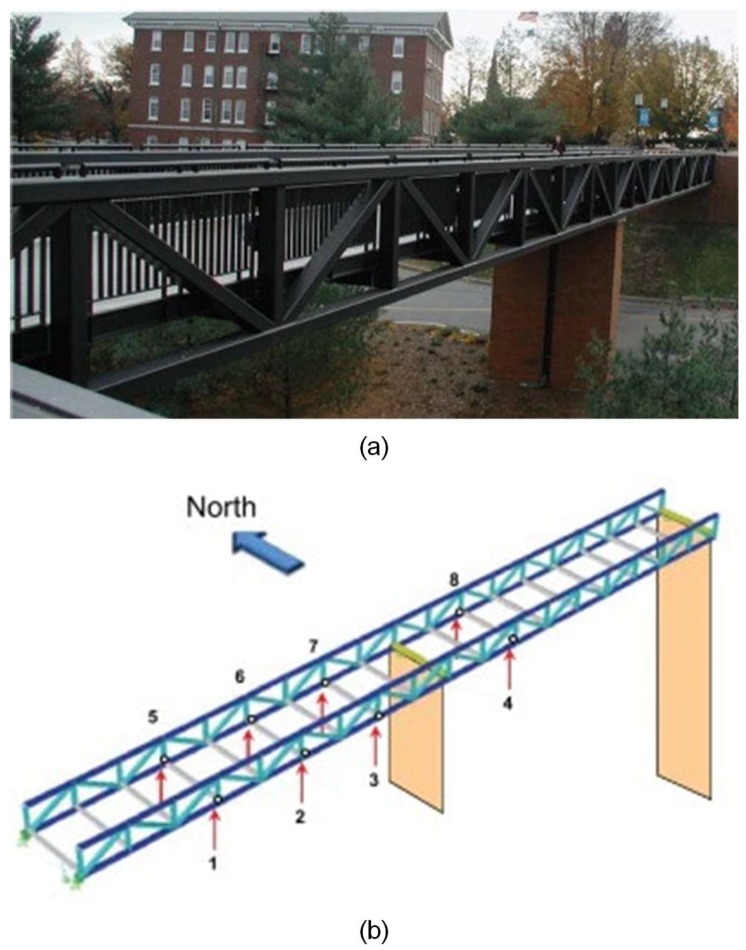
Dowling Hall Footbridge: (**a**) the overview; (**b**) the layout of accelerometers [43].

A vibration-based continuous SHM system with 8 accelerometers and 10 thermocouples has been deployed on the Dowling Hall Footbridge (DHF) at Tufts University in Medford. This footbridge is 44 m long and 3.7 m wide. As shown in Figure 14a, it is a two-span continuous steel frame bridge. The eight uniaxial accelerometers were permanently installed on the underside of the bridge [42] to measure the structural vibration under ambient stimulations, as displayed in Figure 14b. More details about this bridge and its monitoring system can be found in [42,43].

**Figure 15:**
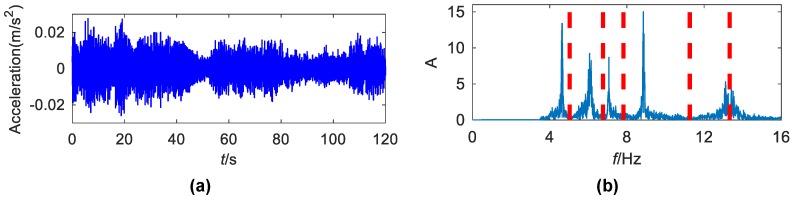
(**a**) The time history of the analyzed acceleration from DHF; (**b**) the detected boundaries in the Fourier spectrum.

**Figure 16:**
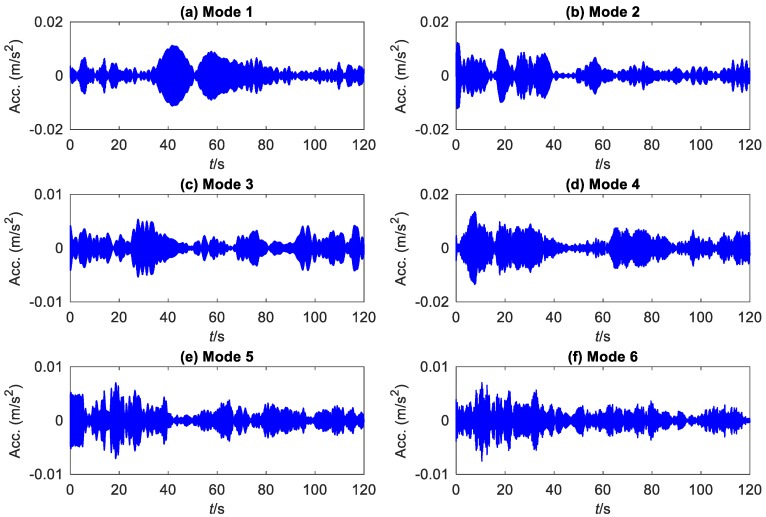
The first six components extracted from the acceleration signal of DHF by EWT (The Acc. represents acceleration).

Data of seventeen weeks have been released to the public. A set of 300-s data was recorded once an hour with a sampling frequency of 2048 Hz. This study analyzes acceleration data collected by accelerometer number 1 (Figure 11b) from 11:30 a.m. to 12:00 a.m. on 30 March 2010. Its time history and detected frequency boundaries are shown in Figure 15. Figure 16 displays the first six components extracted by EWT.

**Figure 17:**
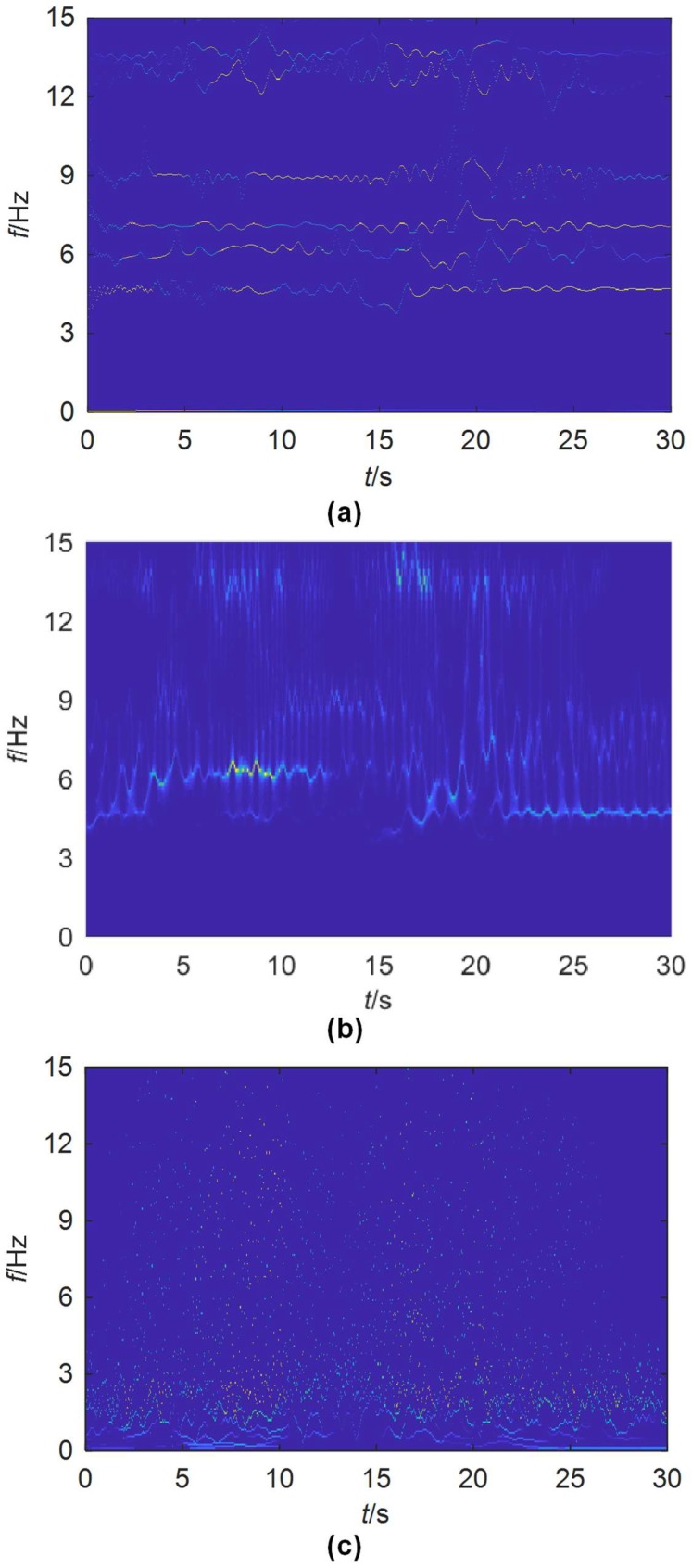
TF planes for the acceleration signal of DHF obtained by: (**a**) EWT; (**b**) SWT; (**c**) EMD.

The instantaneous modal characteristics of the bridge corresponding to the six extracted modes are obtained by the FREEVIB method. The identified instantaneous frequencies are shown in Figure 17, compared with those from SWT and EMD. All the six modes have been clearly separated by EWT, even the two (modes 5 and 6) quite close to each other. However, no clear and reliable modes are extracted by SWT and EMD. That is to say, the frequency resolution of EWT is much higher than that of SWT and EMD, implying that it is better to separate the SHM signals of a civil structure into meaningful mono-components.

**Figure 18:**
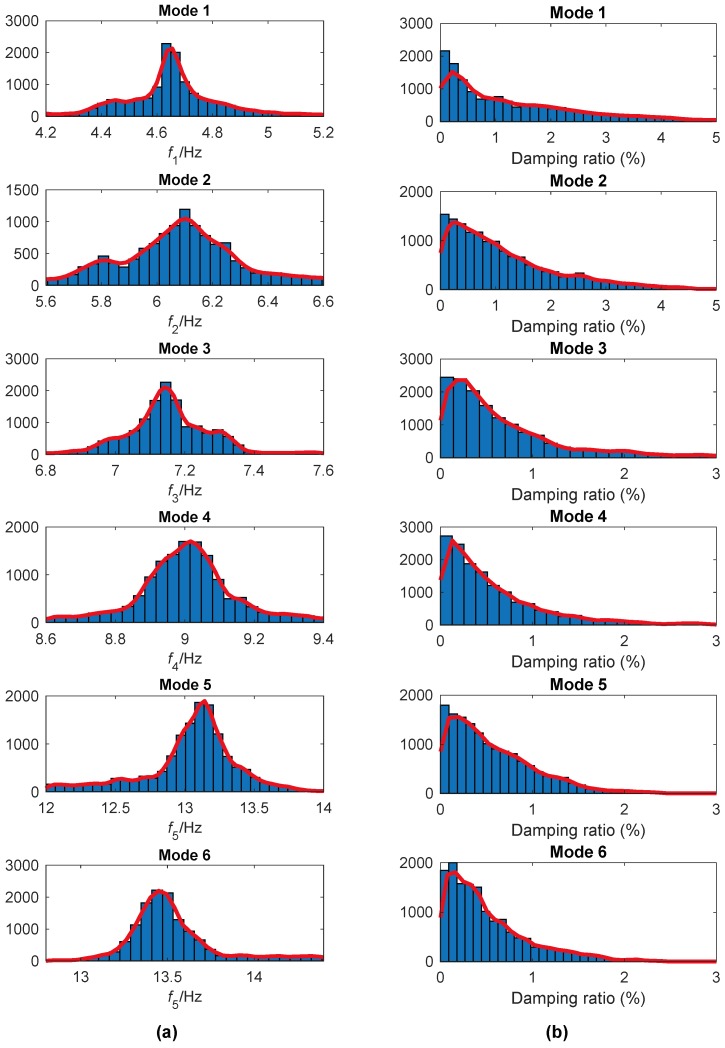
Histograms for the instantaneous modal parameters of DHF identified by EWT: (**a**) Instantaneous frequency; (**b**) damping ratio.

Figure 18 shows histograms of the instantaneous frequencies and damping ratios for the extracted modes obtained by the FREEVIB method. As in the Canton tower case study, the most probable values corresponding to each mode is adopted as the modal parameter of the structure. The modal parameters derived in this way are listed in Table 3 as well as the corresponding CVs. The CVs for the fifth and sixth modal frequencies are much larger than those for others. The reason is that these two modes are more sensitive to disturbances, and the corresponding instantaneous frequencies fluctuate relatively greatly with time, as shown in Figure 14a. Similar to the CVs of the damping ratio for Canton Tower, those of DHF are also very large because of the inherent instability.

**Figure 19:**
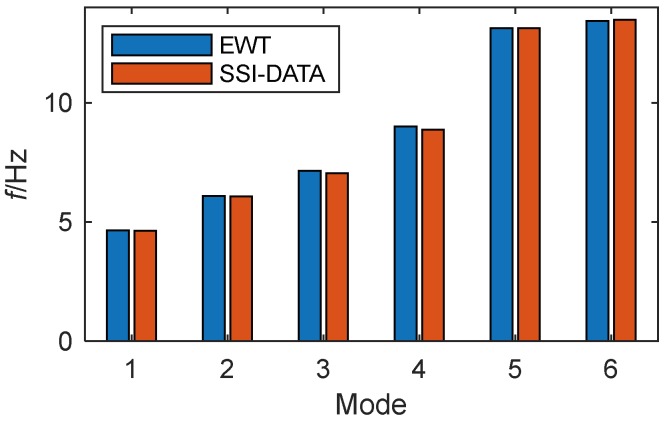
Comparison of modal frequencies for DHF identified by EWT and SSI-DATA.

To verify the modal frequencies identified by the proposed methodology, they are compared with those from a preliminary dynamic test on 4 April 2009 [44], for which the modal parameters were identified by the SSI-DATA method. The comparison bars are shown in Figure 19. The EWT results are extremely close to those determined from the modal test. The closely spaced modes, i.e., the fifth and the sixth ones, are well identified by EWT. In contrast, the SSI-DATA method requires selecting the order carefully.

**Figure 20:**
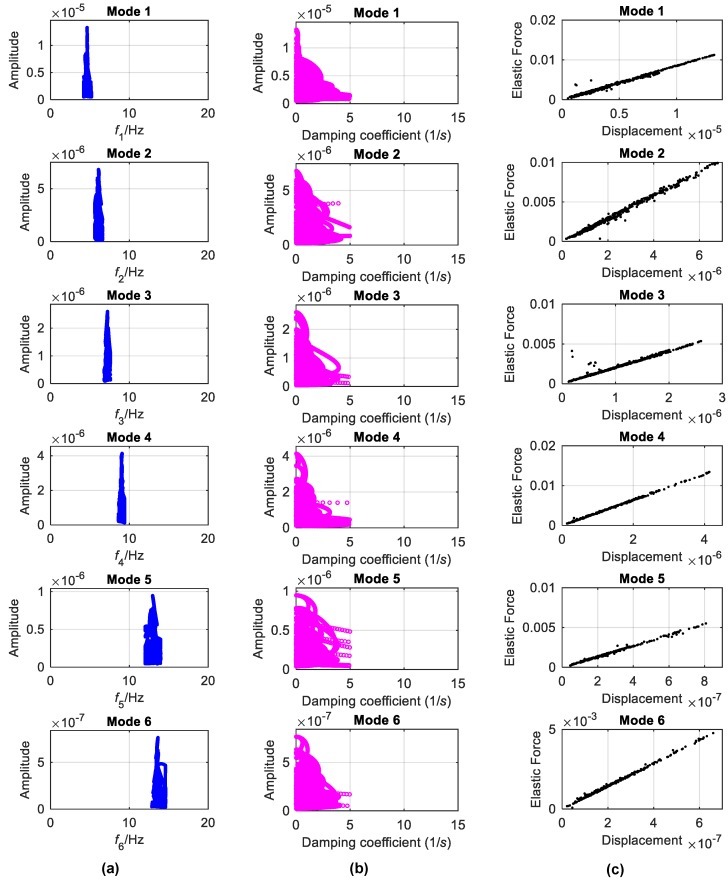
Linear characteristics identified from mono-components of DHF signal: (**a**) Backbones; (**b**) damping coefficients; (**c**) elastic forces.

Using the extracted mono-components, the backbones, damping curves and elastic forces corresponding to each mode of the bridge are also analyzed by the FREEVIB method (Figure 20). The backbones can be considered as vertical lines, implying that the identified instantaneous frequencies do not vary with the vibration amplitude. Though the damping coefficients are quite decentralized, they also do not change evidently with the amplitude. It is deduced that the DHF can be considered as a linear system in the analyzed condition, which is further validated by the identified elastic-force characteristics shown in Figure 20c.

## 6. Discussion

Due to the large-scale and complexity, together with the intricate structure-load interactions, SHM signals acquired from civil structures are complex but contain rich information about the structural condition. For structural evaluation, it is desirable to extract instantaneous structural features from these complex signals. EWT is a promising tool because it is an advanced TF method that combines the merits of both EMD and WT. However, in the processing of real SHM signals, the original EWT method proposed by Gilles [17] is very susceptible to noises, leading to false signal decomposition. Another problem is that after separating the signals, the traditional way is to convert the mono-components into free decaying vibration function subjected to ambient vibrations. Consequently, the time-varying features of the structure are ignored. This paper proposes a systematical procedure to extract the instantaneous structural features based on the improved EWT.

The accuracy and boundary detection capability of the scale-space method using the Otsu’s algorithm are demonstrated by both the numerical study and experimental study on real civil structures (Figure 2, Figure 4, Figure 5, Figure 6, Figure 8, Figure 10, Figure 15 and Figure 17). Its immunity to noises is also tested by the synthetic signals with different levels of noises. Compared with EMD and SWT, the proposed scale-space EWT can extract more accurate modes. All the concerned modes were separated from the studied signals, without extra ones. However, from Figure 4, Figure 10 and Figure 17, some redundant modes that are difficult to be interpreted are produced by EMD. The reason is that EMD forces the extraction of IMFs through an ad-hoc process even if the initial components are not. The frequency resolution of EWT is much higher than that of traditional WT methods including the SWT, a smart utilization of the output of the classical WT (Figure 4, Figure 10 and Figure 17). Even the closely spaced modes can still be well discriminated by the scale-space EWT.

By applying FREEVIB to the mono-components extracted from the vibration signals of civil structures, the structural features, including the instantaneous modal parameters and the linearity characteristics, are derived. In the case study, the obtained modal parameters are proved to be reasonable by comparing with those identified by the traditional methods such as SSI-DATA and EFDD. In contrast with the usual way that considers the extracted modes as free decaying vibrations, the FREEVIB retains the instantaneous features of these parameters. It implies that the time-varying performance of structure can be tracked, which provides a new perspective for SHM-based structural condition assessment. For example, if there is any anomaly in the structure, these characteristics or their derivations such as the parameters for their statistical distributions may changes correspondingly. In addition, the linearity of the structural system can also be judged by the FREEVIB results.

Spectra instead of the Fourier one, such as the standardized autoregression power spectrum [29] and the pseudo-spectrum obtained by the multiple signal classification approach [30,31], have been used in some studies to reduce the disturbance of noises on the boundary detection in EWT. Nevertheless, it may not be so convinced that no useful structural information is overlooked. This study still uses the Fourier spectrum to build the wavelet filter bank, though it is susceptible to noises. For this study, the improvement of EWT lies in the boundary detection method, or the definition of boundaries. The Fourier spectrum is transformed to the scale-space, and the boundaries are those corresponding to scale-space curves above a certain length.

In this study, some noises embedded in the signal were removed at the beginning of the EWT procedure, and the results are as expected. However, the denoising method is trail-based. A thorough investigation on how to remove noises effectively is necessary in the future. The best spectrum segmentation method to extract different modes, including but not limited to spectra to be used, and algorithms for boundary definition, is still an open question to be addressed.

Though the instantaneous structural characteristics of the civil structures are obtained by the proposed methodology, how to take advantages of these results in the structural condition assessment is also another direction to explore.

## 7. Conclusions

This paper proposes a systematic methodology to extract instantaneous features about the structural condition from SHM signals of civil structures. The signal is decomposed into individual components using a scale-space EWT method first. Subsequently, the FREEVIB method is applied to the extracted mono-components to obtain the time-varying structural characteristics.

The scale-space EWT means an EWT method detecting the frequency boundaries for the wavelet filter bank in the scale-space representation of the traditional Fourier transform. The boundaries are defined as those with scale-space curves above a certain threshold. To find the threshold, the Otsu’s method is adopted in this study. The scale-space EWT aims to improve the original EWT method proposed by Gilles [17] that is sensitive to noises and requires a predefined boundary number.

For modal identification of civil structures, the traditional way to utilize the decomposed signal components is to regard them as free decaying vibrations. However, the time-varying effect of the modal parameters is ignored consequently. This paper employs the FREEVIB method to process the EWT-extracted mono-components. By this means, the instantaneous modal parameters are obtained. Moreover, this method can analyze the linear characteristics of the structure based on backbones, damping coefficients and elastic forces.

Both numerical and experimental studies are conducted to validate the proposed method. Different levels of noises are added to the simulated signals to test its immunity. The performance of the scale-space EWT in boundary detection, and the accuracy of the extracted instantaneous frequencies are verified by comparing with the results of EMD and SWT. Real SHM signals from a high-rise building and a footbridge are analyzed. The instantaneous modal parameters are reasonable after being compared with results in the previous studies.

The proposed signal processing procedure is effective to identify the instantaneous modal parameters and obtain the linearity characteristic of civil structures. Moreover, this method can deal with the high-level noisy signals. Studies on the optimal spectrum segmentation, and the utilization of the obtained instantaneous features in structural condition assessment, will be carried out in the future.

## Figures and Tables

**Table 1:** Comparison of EWT, SWT, and EMD using the simulated signal.

Modes	Methods	Frequency (Hz)		CV	Damping Ratio (%)		CV
		Analyzed	Theoretical		Analyzed	Theoretical	
1	EWT	1.01	1	0.03	0.41	0.80	1.37
	SWT	1.01		0.02	0.35		0.82
	EMD	1.00		0.02	0.33		0.76
2	EWT	3.06	3	0.01	0.26	0.27	0.64
	SWT	3.09		0.01	0.28		1.51
	EMD	3.08		0.27	0.70		1.79
3	EWT	6.00	6	0.05	0.28	0.13	1.90
	SWT	6.00		0.32	2.44		1.45
	EMD	6.09		0.52	1.89		0.98

**Table 2:** Modal parameters identified from signal mono-components of Canton Tower.

Mode	Frequency (Hz)	CV	Damping Ratio (%)	CV
1	0.0948	0.05	0.78	0.84
2	0.3659	0.06	0.34	1.04
3	0.4847	0.04	0.33	1.03
4	0.7983	0.02	0.23	1.24
5	1.1610	0.08	0.15	1.41

**Table 3:** Modal parameters identified from mono-components of DHF.

Mode	Frequency (Hz)	CV	Damping Ratio (%)	CV
1	4.66	0.05	0.21	1.24
2	6.10	0.09	0.29	0.90
3	7.16	0.02	0.27	1.22
4	9.02	0.04	0.13	1.44
5	13.14	0.40	0.20	1.34
6	13.45	0.49	0.16	1.19
